# Chromium removal from aqueous solution by a PEI-silica nanocomposite

**DOI:** 10.1038/s41598-018-20017-9

**Published:** 2018-01-23

**Authors:** Keunsu Choi, Soonjae Lee, Jin Ock Park, Jeong-Ann Park, So-Hye Cho, Seung Yong Lee, Jun Hee Lee, Jae-Woo Choi

**Affiliations:** 1 School of Energy and Chemical Engineering, Ulsan National Institute of Science and Technology, Ulsan, 44919 Republic of Korea; 2 Department of Earth and Environmental Sciences, Korea University, 145, Anam-ro, Seongbuk-gu Seoul, 02841 Republic of Korea; 3 Materials Architecturing Research Center, Korea Institute of Science and Technology, Hwarang-ro 14-gil 5, Seongbuk-gu Seoul, 02792 Republic of Korea; 4 Center for Water Resource Cycle Research, Korea Institute of Science and Technology, Hwarang-ro 14-gil 5, Seongbuk-gu Seoul, 02792 Republic of Korea; 5 Division of Nano & Information Technology, KIST School, Korea University of Science and Technology, Hwarang-ro 14-gil 5, Seongbuk-gu Seoul, 02792 Republic of Korea; 6 Division of Energy & Environment Technology, KIST School, Korea University of Science and Technology, Hwarang-ro 14-gil 5, Seongbuk-gu Seoul, 02792 Republic of Korea

## Abstract

It is essential and important to determine the adsorption mechanism as well as removal efficiency when using an adsorption technique to remove toxic heavy metals from wastewater. In this research, the removal efficiency and mechanism of chromium removal by a silica-based nanoparticle were investigated. A PEI-silica nanoparticle was synthesized by a one-pot technique and exhibited uniformly well-dispersed PEI polymers in silica particles. The adsorption capacity of chromium ions was determined by a batch adsorption test, with the PEI-silica nanoparticle having a value of 183.7 mg/g and monolayer sorption. Adsorption of chromium ions was affected by the solution pH and altered the nanoparticle surface chemically. First principles calculations of the adsorption energies for the relevant adsorption configurations and XPS peaks of Cr and N showed that Cr(VI), [HCrO_4_]^−^ is reduced to two species, Cr(III), CrOH^2+^ and Cr^3+^, by an amine group and that Cr(III) and Cr(VI) ions are adsorbed on different functional groups, oxidized N and NH_3_^+^.

## Introduction

The term “heavy metals” refers to metals with specific gravities of approximately 4.0 or higher, which are distributed mostly in rock and soil. Heavy metals produced by smelting processes are used for various purposes, such as batteries, blood pressure gauges, thermometers, building blocks, and in the machinery industry^1–4^. Ions of heavy metals discharged from a wide range of industrial processes from anthropogenic sources have become serious environmental and human health problems^4,5^.

Among the heavy metals, chromium is used in various industrial fields, such as the metallurgical, steel, tannery and cement industries, which are also major emission sources^6^. Chromium discharged from industries is most commonly in the form of trivalent (Cr(III)) or hexavalent (Cr(VI)) chromium in water^7^. Cr(III) is an essential human dietary element and, Cr(VI) occurs naturally in nature from the erosion of chromium deposits. These can also be produced by anthropogenic emissions, such as industrial processes. However, Cr(III) and Cr(VI) have significant differences related to their characteristics, including toxicological and biological properties^6^. Cr(VI) is considered to be a very harmful compound because of its characteristics of being water soluble and carcinogenic^8^. In contrast, Cr(III) is less soluble and relatively non-toxic over a wide pH range. Due to its toxicity, the US EPA and the EU established the permissible emission standards for Cr(III) and Cr(VI) as a total chromium amount of 0.1 mg/L in 1991^6^.

For these reasons, studies on reducing the amount of chromium below the effluent water quality standard are very important issues. Various techniques have been studied to purify chromium-contaminated water, including ion exchange^9,10^, membrane processes^11^, electrodialysis^12^, precipitation^13^, bio-sorption^14^, photocatalytic degradation^15^, and adsorption^16,17^. In addition, Cr(III) can be easily precipitated from the aqueous phase by hydroxide or oxide foams. Therefore, the chemical reduction of toxic Cr(VI) to Cr(III) by precipitation is a common method for the treatment of wastewater with Cr(VI)^18,19^. However, unfortunately, this method increases the already huge production of sludge, resulting in limited field applications. Compared to other techniques, adsorption is the most promising technology due to its high efficiency, ease of operation, and economic advantages^20^. Moreover, the greatest advantage of the adsorption method is that by-products, such as sludge, are not generated.

Various adsorbents for Cr(VI) removal, such as activated carbons, zeolites, clays, etc., have been investigated^21–27^. However, silica is superior to the other materials mentioned above because of its high porosity, large surface area and great number of functionalities^28,29^. For these reasons, a great amount of research on silica as Cr(VI) adsorbents has been carried out^30–38^. Usually, the preparation of silica adsorbents consists of two stages, including the synthesis of porous silica and the attachment of functional groups for Cr(VI) adsorption onto the silica surface.

In this study, we investigated a PEI-silica nanoparticle, which was prepared by a simple one-step synthetic method, as an adsorbent to remove toxic chromium in solution. Furthermore, the adsorption capacity for chromium was extensively investigated, and its chromium adsorption mechanism was clarified, which may be applicable for silica-based materials in general.

## Results and Discussion

### Characteristics of the PEI-silica Nanoparticles

**Figure 1:**
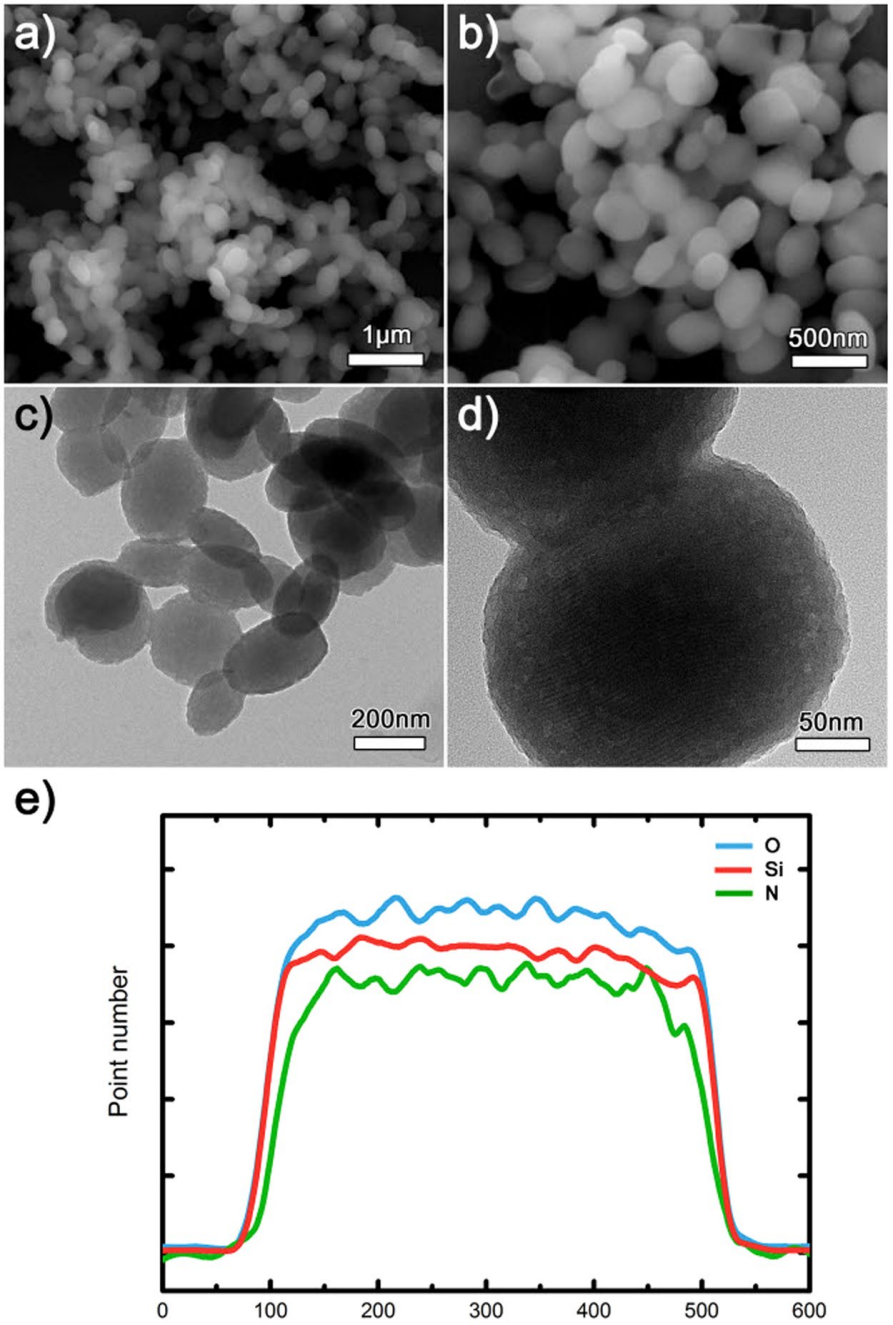
Morphological analyses of the PEI-silica nanoparticles with (**a**,**b**) SEM and (**c**,**d**) TEM. (**e**) Line scanning result (rescaled for comparison) of a nanoparticle in (**d**).

The electron microscopy analysis by SEM and TEM indicates that the particles have near spherical and elliptical morphologies (Fig. 1). The size of the particles is in the range of approximately 200–300 nm, as also shown in Fig. 1. The line scan result by TEM reveals that silicon and nitrogen atoms are uniformly distributed. This indicates that the PEI polymers are uniformly dispersed in the silica particles. Also, the TEM mapping result of nitrogen atoms supports the uniform dispersion of PEI polymers. (Fig. S1 in Supporting Information).

**Figure 2:**
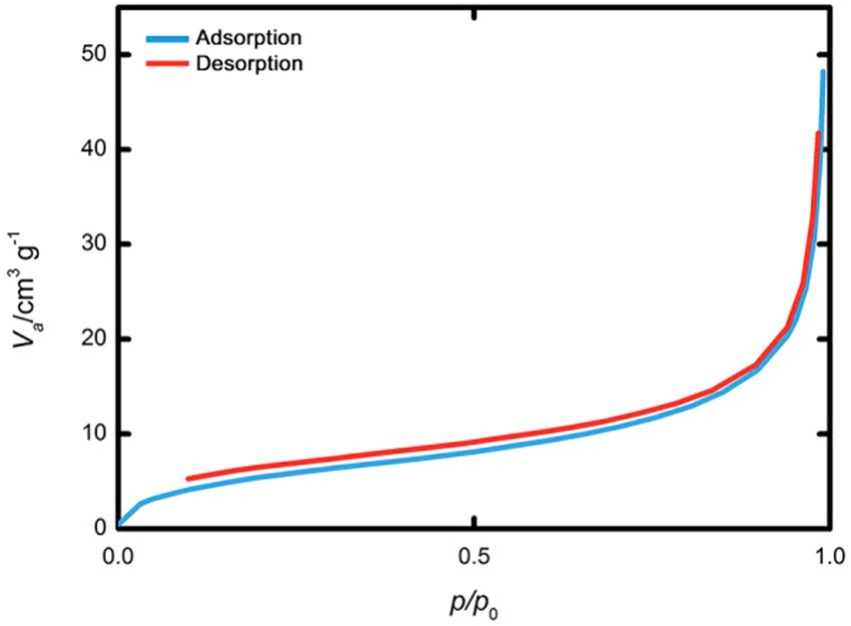
N_2_ adsorption-desorption isotherm graph of silica nanoparticles at 77 K.

The surface areas and pore volumes of the silica nanoparticles were analyzed by the nitrogen (N_2_) adsorption-desorption technique at 77 K. As shown in Fig. 2, the silica nanoparticles exhibited a characteristic type III BET isotherm graph, which indicates that the PEI-silica nanoparticles are essentially non-porous. This result agrees with the very small BET surface areas and pore volumes of the materials in Table 1. Therefore, amine groups exposed on the surface participate in the adsorption of Cr(VI).

**Table 1:** Physical characteristic of the PEI-silica nanoparticles from the N_2_ adsorption-desorption analysis.

	BET surface area [m^2^/g]	Mesopore volume [cm^3^/g]
Silica nanoparticles	21.60	0.07

**Figure 3:**
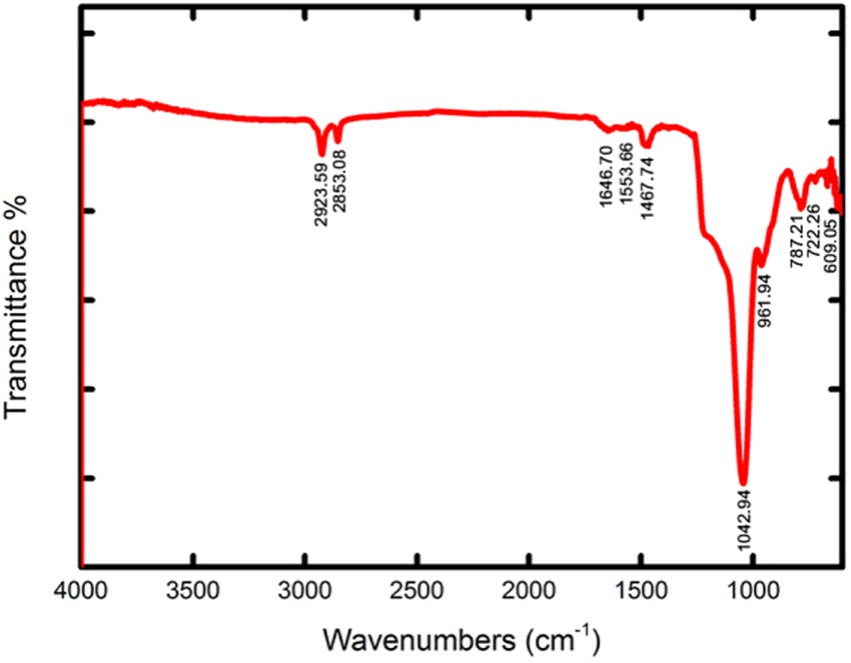
FT-IR spectrum of the PEI-silica nanoparticles.

In the FT-IR results, the strong absorption band at 1042 cm^−1^ is due to stretching asymmetric vibrations of the Si-O-Si band (Fig. 3)^39^. The absorption peak at 787 cm^−1^ is assigned to the bending vibrations of Si-O-Si group, while the absorption peak at 609 cm^−1^ are ascribed to the symmetric stretching vibrations of the Si-O-Si band^40^. It is thought that the absorption peak at approximately 961 cm^−1^ is related to the Si-O- groups. The existence of PEI is confirmed by the presence of absorption features of PEI at 1553–1646 cm^−1^. This peak is assigned to the bending of N-H_2_^41,42^. The two bands at 2853 cm^−1^ and 2923 cm^−1^ agree well with the C-H_2_ absorption bands^43,44^. The peak at 1468 is assigned to symmetric bending mode of NH^3+^ in the Si-O- … NH^3+^ groups^45,46^.

**Figure 4:**
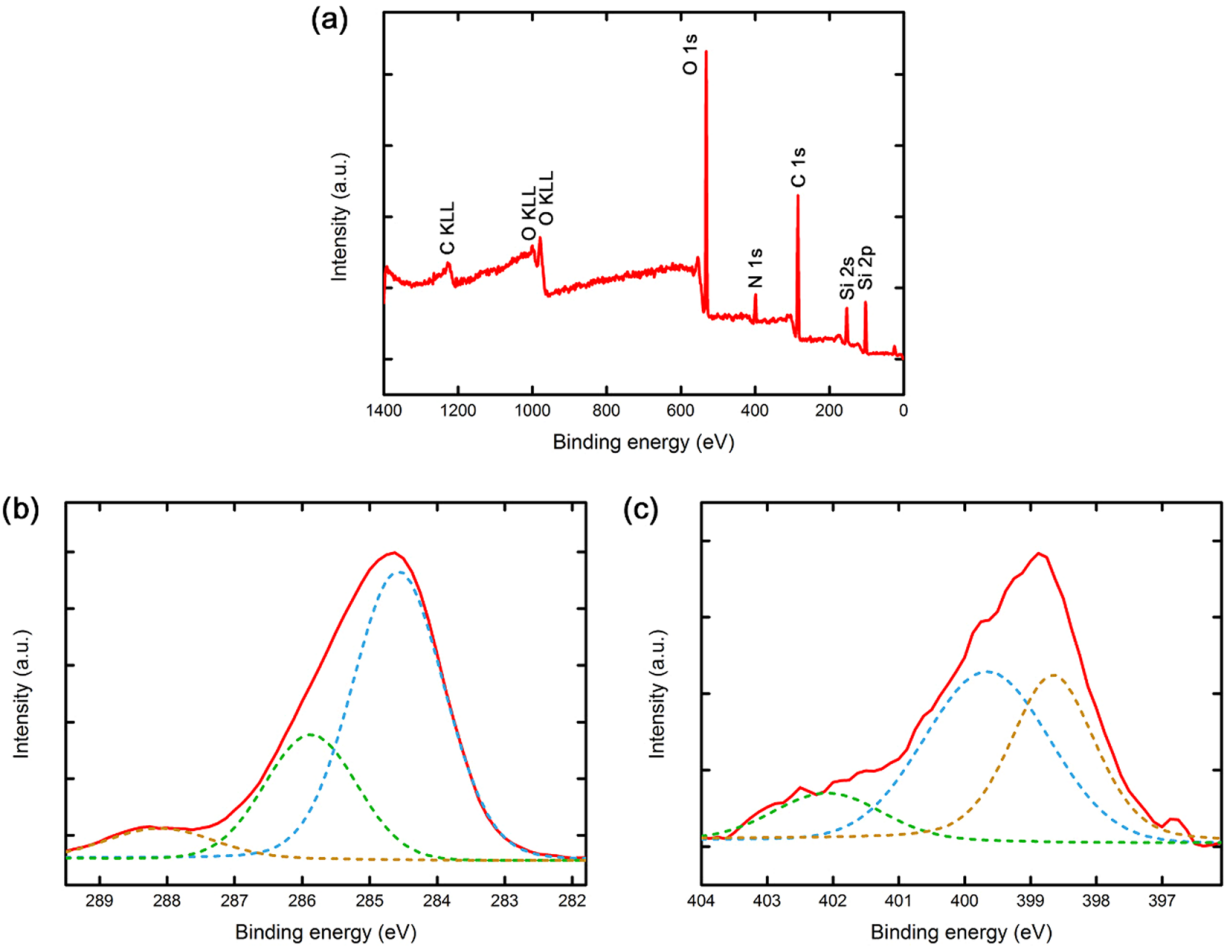
(**a**) XPS spectra of the PEI-silica nanoparticles and (**b**) curve-fitting for C 1 s and (**c**) N 1 s.

For further elemental analysis of the PEI-silica nanoparticles, an X-ray photoelectron spectroscopy (XPS) measurement was performed. The XPS spectrum of Si atoms in Fig. 4 consists of peaks with binding energies of 153 eV (Si 2 s) and 103 eV (Si 2p)^46^. Additionally, the C 1 s and O 1 s peaks in the PEI-silica nanoparticles were detected near 284 eV and 532 eV, respectively. The C 1 s peak at approximately 285 eV typically corresponds to C-C and C-N bonds^47,48^. As shown in Fig. 4(b), an asymmetric peak in the spectrum of C 1 s consists of three peaks with binding energies of 284.58, 285.87 and 288.11 eV. These are considered to originate from the conjunction bonding with adjacent N atoms by PEI^49^. More importantly, the XPS spectrum in Fig. 4(c) at approximately 400 eV indicates the presence of N atoms with three different binding energies. The binding energies of N 1 s are 398.66, 399.66 and 402.09 eV. It is noticeable that the peak area portion of protonated amine groups, NH_3_^+^, at 402.09 eV is only 11.7%, while most of the peak, 88.3%, originates from primary amines, secondary amines and their bonds with oxygens^50^. It was reported that the content of primary amines, secondary amines, and tertiary amines of the PEI used in this research is 44%, 33%, 23%, respectively^51^. The content of PEI was evaluated by measuring the mass change after burning-out at 480 °C for 12 hrs. The portion of PEI ranges from 37–40 wt%. Also, TG analysis showed similar PEI content, 42%. (Fig. S2 in Supporting Information).

### Chromium Adsorption of the PEI-silica Nanoparticles

The applicability of the PEI-silica nanoparticles for the removal of Cr from aqueous solution was investigated by conducting equilibrium batch experiments. The reusability of PEI-silica was also validated through successive regeneration and reuse tests (Exp. S1 in Supplementary Information). Additionally, the effect of the solution pH on the chromium adsorption was investigated by adjusting the initial pH from 2 to 4. PEI-silica nanoparticles showed significant removal of chromium, but the pure nanosilica, in the control test, showed negligible chromium removal (data not shown) despite of a very large BET surface area, 1379.6 m^2^ g^−1^. This indicated that the chromium removal resulted from the PEI group on the nanoparticle.

**Figure 5:**
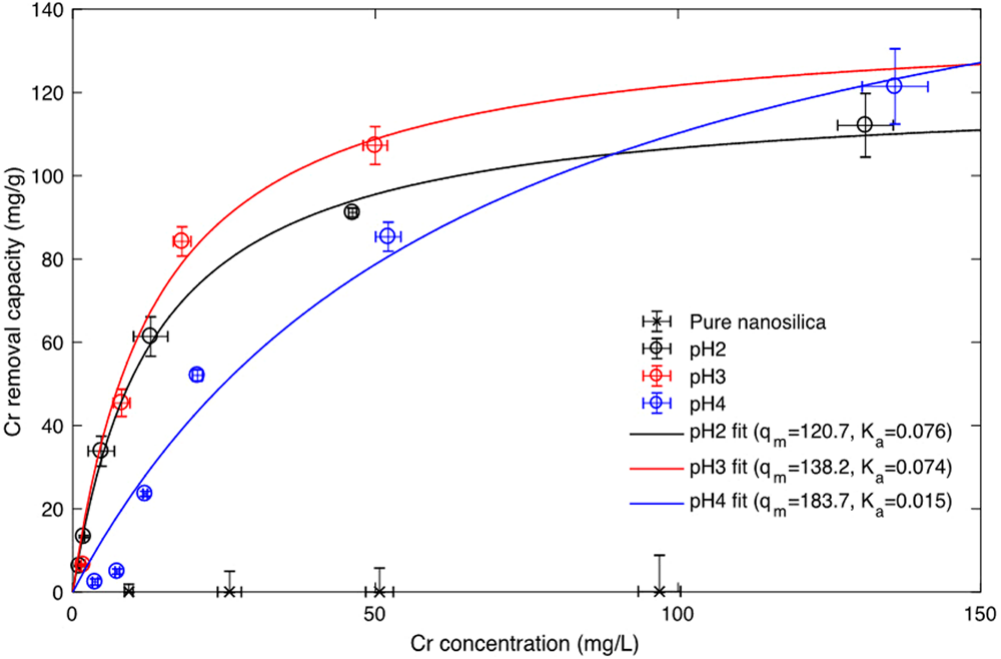
Cr adsorption isotherm of pure nanosilica and PEI-silica nanoparticle under pH 2, 3 and 4 and its fitting with the Langmuir adsorption model.

The chromium adsorption isotherms at the different pH levels are shown in Fig. 5. The PEI-silica nanoparticle exhibited non-linear and pH-dependent adsorption isotherms for the removal of chromium. The adsorption isotherms are well-fitted with the Langmuir adsorption model. The isotherms for pH values of 2, 3 and 4 exhibited high maximum adsorption capacities of 120.7, 138.2 and 183.7 mg/g, respectively. The isotherms of initial pH values of 2 and 3 showed similar rate of adsorption (similar K_a_), but the isotherm of the pH of 4 showed a different trend than the others, with a much lower K_a_. This shows that the chromium removal capacity of the PEI silica nanoparticle increased in the higher pH conditions, but the chromium adsorption reaction was different at higher pH conditions. The chromium adsorption capacity of PEI-silica nanoparticles was higher than those presented in previous research related to chromium removal using adsorbents, including surface-functionalized mesoporous iron oxyhydroxide (25.05 mg/g), magnetite (4.84 mg/g), nanocarbon (27.29 mg/g), coconut-derived activated carbon (13.9 mg/g), algal bloom residue-derived activated carbon (155.52 mg/g), and metal organic frameworks (48 mg/g)^52–56^.

### Reduction of Cr(VI) Ion by N-containing PEI-silica Nanoparticle

**Figure 6:**
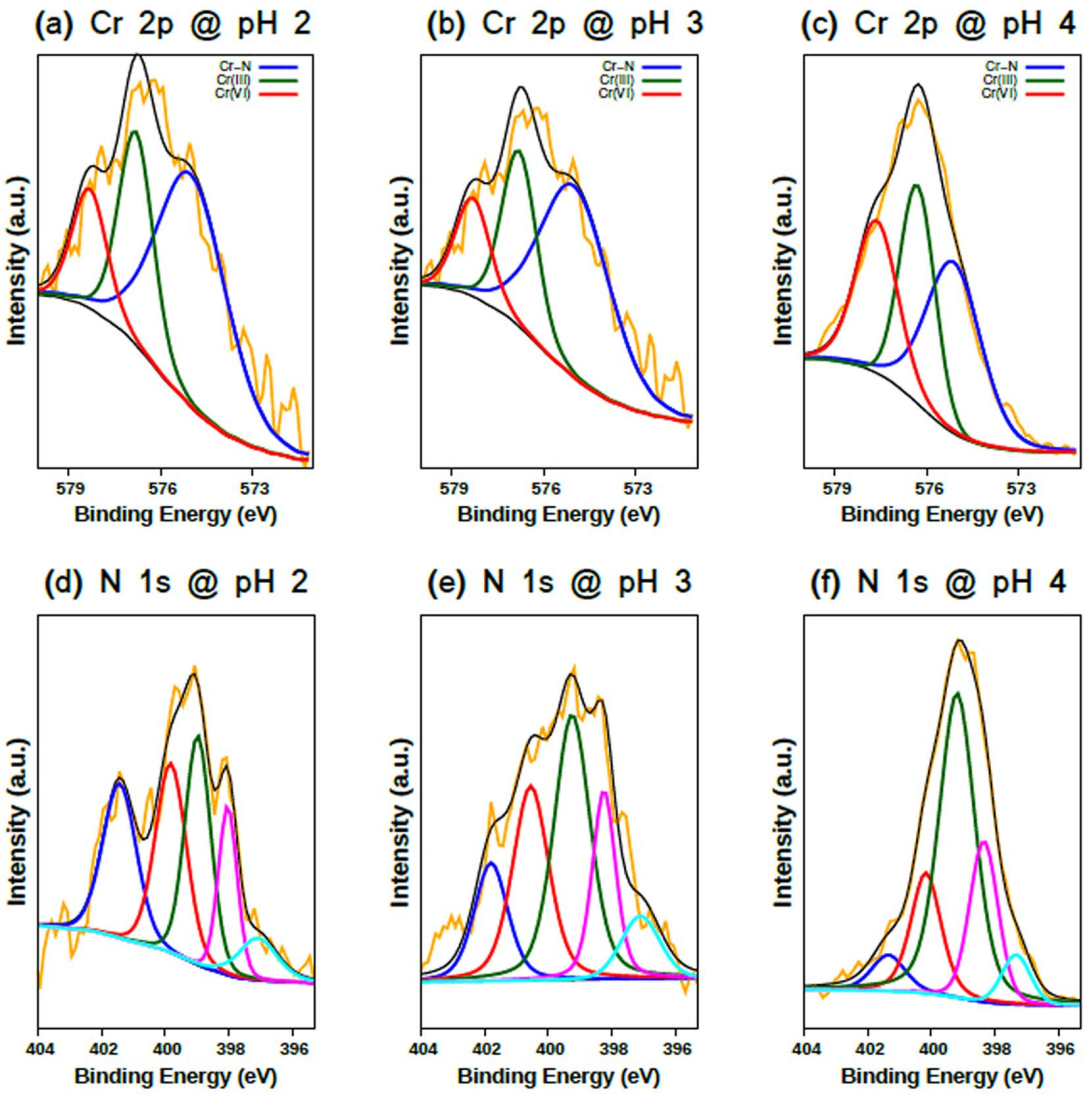
(**a**)–(**c**) Cr 2p XPS spectra and (**d**)–(**h**) N 1 s XPS spectra of the PEI-silica nanoparticle at pH 2–4 after Cr adsorption.

The effect of pH on the adsorption of Cr in the solution was investigated by conducting the batch adsorption test at different initial pH levels while maintaining the other conditions as constant. The PEI-silica nanoparticles give the highest adsorption capacity of 183.7 mg/g at a pH of 4 and decreases monotonically at lower and higher pH values. To identify the adsorption site and the species of the adsorbed Cr ions, we analyzed each component in PEI samples in the range of pH 2 to 4 using XPS. Figure 6 shows N XPS 1 s spectra (a-c) and Cr 2p XPS spectra (d-f) of PEI samples after Cr adsorption. In the Cr 2p_2/3_ XPS spectra, three peaks are shown at approximately 575.0, 576.5, and 578.0 eV, which are assigned to Cr, CrOH, and Cr(VI), after Cr adsorption. The presence of Cr(III) ions indicates that the Cr(VI) ion is reduced in water by a N-containing functional group. Considering that the amount of trivalent chromium decreases as the pH value increases, the −NH functional group can be thought to participate in the reduction reaction of Cr(VI), since the amount of the −NH functional group shows the opposite trend to that of Cr(III) depending on the pH value. This reaction resembles Jones oxidation, which indicates the reduction and oxidation reactions between hydroxyl group and Cr ion, except for the −NH group.

The ratio between Cr and CrOH also depends on the pH value, and the amount of Cr decreased with the pH value, while that of CrOH increased. According to the poubaix diagram, Cr^3+^ and CrOH^2+^ are dominant in the low and high pH values in the range of pH 2 and 4, respectively^57^. Both species of Cr(III) ions are adsorbed on PEI by making covalent bonds with N atoms, directly. On the other hand, Cr(VI) ions are adsorbed on the NH_3_^+^ functional group through hydrogen bonding. These adsorption behaviors will be discussed in terms of theoretical calculations in the next session. The amount of Cr(VI) ions increased with the pH value, while that of Cr(III) decreased, as mentioned above. This result indicates that the reduction reaction of Cr(VI) depends on pH value.

### First Principles Calculations

**Figure 7:**
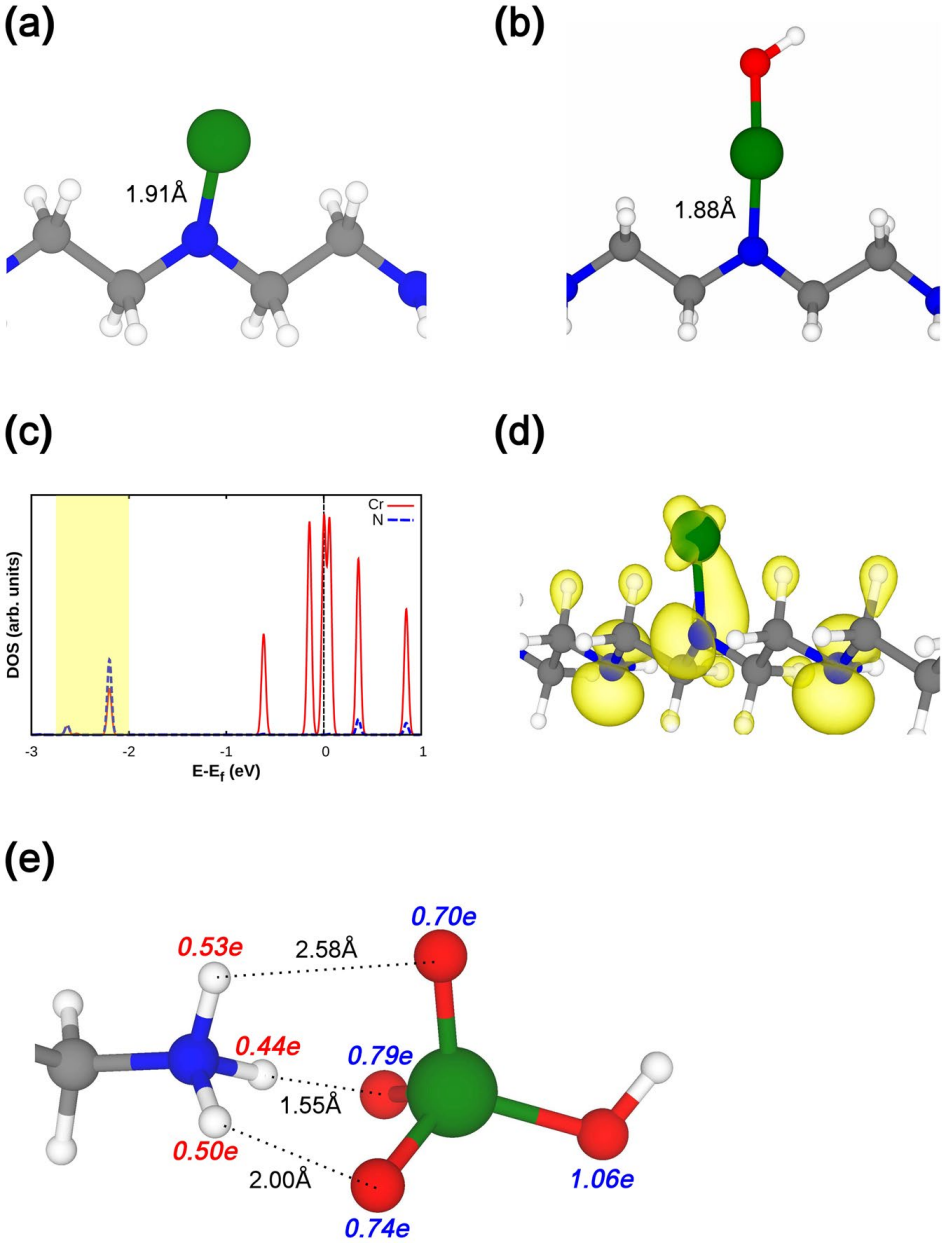
Cr(III), (**a**) Cr^3+^ and (**b**) [Cr(OH)]^2+^, makes a covalent bonding with oxidized secondary amine group, − N =. (**c**) Partial density of state (PDOS) shows orbital hybridization between Cr d-orbital and N p-orbital in the energy range −2.75 to −2.00 eV (yellow shaded region). (**d**) The partial charge density in the same energy range also shows the orbital hybridization between Cr d-orbital and N p-orbital. The isosurface level of partial charge density is 0.1e/Å^3^. (**d**) Cr(VI), [HCrO_4_]^−^, is adsorbed on protonated primary amine, NH^3+^, by hydrogen bonding. The amount of positive (negative) charges on each atom is indicated by red (blue) number. White, grey, blue, red, and green balls represent hydrogen, carbon, nitrogen, oxygen, and chromium atom, respectively.

We carried out first principles calculations to identify the adsorption mechanisms between Cr ions and adsorption sites in the PEI-silica nanoparticles. We investigated combinatorial configurations of three adsorbates (Cr, CrOH, and HCrO_4_) and four adsorption sites (−N =, −NH−, −NH_2_, −NH_3_) based on the XPS spectra. The calculation results show that Cr(III) and Cr(VI) ions favor different adsorption processes. Both Cr(OH)^2+^ and Cr^3+^ ions bind to N atoms directly by forming covalent bonds, of which the bond lengths between Cr and N atoms are 1.88 Å and 1.90 Å, as shown in Fig. 7(a,b), respectively. In Fig. 7(c), the density of states (DOS) for the Cr-N configuration is given and the orbital hybridization of the two atoms is given in the energy range of −2.75 eV and −2.00 eV with respect to the fermi energy, which corresponds to the region painted in translucent yellow. We further calculated the charge density over that energy range and the shape of the partial charge density plot indicates the orbital hybridization of the d-orbital of Cr and the p-orbital of N in Fig. 7(d). On the other hand, Cr(VI) binds to −NH_3_^+^ through hydrogen bonding, as shown in Fig. 7(d). The bond lengths between three O atoms in Cr(VI) and three H atoms in −NH_3_ are 1.55 Å, 2.00 Å, and 2.58 Å, respectively.

## Conclusions

This research aimed to develop a novel nanoparticle for the removal of chromium from solution. Equilibrium batch tests were conducted to examine the adsorption capacity of the developed adsorbent. The maximum adsorption capacity for chromium was found to be 183.7 mg/g. Experiments with various initial pH values (2 to 4) were conducted under controlled conditions and found that the removal efficiency of the PEI-silica nanoparticles was affected by the solution pH, and the highest removal efficiency of chromium occurred at a pH of 4. The amine group participates in the reduction reaction of Cr(VI) to Cr(III) by oxidizing itself. The reduced Cr(III) ions, Cr^3+^ and CrOH^2+^, are adsorbed on oxidized N by covalent bonding. On the other hand, Cr(VI), HCrO_4_^−^, is adsorbed on a protonated primary amine, NH^3+^, by hydrogen bonding. These results should help improve the understanding of the chromium adsorption mechanisms from industrial wastewater in other forms of nanoparticles containing amine groups.

## Materials and Methods

### Materials

All chemicals were obtained from the indicated suppliers and used without further purification. Tetraethyl orthosilicate (TEOS, 99.999% metal basis), hexadecyltrimethylammonium bromide (CTAB H5882, >98%) and branched polyethyleneimine (PEI, M_W_ = 800) were purchased from Sigma-Aldrich. Cr(VI) solutions were prepared by diluting Cr standard solution (Kanto Chemical, Tokyo, Japan) with deionized water. In addition, all vials and tools were cleaned with ethanol three times and deionized water three times before they were used.

### Synthesis of the PEI-silica Nanoparticle

PEI-silica nanoparticles were synthesized by a one-pot technique. First, 0.656 g of CTAB were dissolved in 180 mL of distilled water and 8.5 mL of PEI were dissolved in 20 mL of ethanol as a shape and pore former. The PEI solution was mixed with the CTAB solution with stirring at 800 rpm for 30 min. Afterward, 4.2 mL of TEOS, as the silica precursor, were added using a micropipette. The solution was stirred for 12 hrs, before all final products were washed by centrifugation with distilled water three times. Finally, after drying at 70 °C overnight, we obtained 200–300 nm nanoparticles. To calculate the content of PEI in PEI-silica nanoparticles, they were calcined at 480 °C in air after 2 hr drying at 100 °C. The weight change after calcination was measured.

### Synthesis of the Pure Nanosilica

For a control experiment, pure nanosilica was prepared by calcination of the PEI-silica nanoparticles. To remove the PEI polymer, they were calcined calcined at 480 °C in air for 12 hrs.

### Characterization of PEI-silica Nanoparticles

The particle sizes and morphologies were observed by scanning electron microscopy (SEM, FEI Inspect F50, AP-tech Company) and transmission electron microscopy (TEM, Tecnai G2 F20, FEI Company). FT-IR spectra were obtained by employing a Nicolet iS 10 FT-IR Spectrometer (Thermo Scientific) over the range of 4000–600 cm^−1^. N_2_ adsorption-desorption analysis was performed using a surface area analyzer (BEL-SORP-max, BEL Japan Inc., Japan). Prior to analysis, the materials were degassed at 100 °C for 2 h to obtain pure PEI-silica materials. The specific surface area was determined from the linear part of the Brunauer-Emmett-Teller (BET) plot (P/P_0_ = 0–1). The total pore volume was evaluated from the adsorbed amount at a relative pressure of ~0.99. Elements in the PEI-silica nanoparticles were analyzed by X-ray photoelectron spectroscopy (XPS) on a PHI 5000 VersaProbe Ulvac-PHI (Physical Electronics, Inc.) with an Al X-ray monochromatic source of K_α_ (1486.6 eV energy at 24.5 W). The binding energy was referenced to the C 1 s line at 284.6 eV from adventitious carbon.

### Batch Experiments for Chromium Removal

Removal of chromium was subjected to equilibrium batch tests. The chromium adsorption test was conducted in 50 mL a conical tube containing 0.02~0.03 g of PEI-silica nanoparticles by adding 50 mL of chromium solutions with concentrations of 10, 20, 50, 100 and 200 mg/L. To investigate the effect of the solution pH on chromium removal, the batch experiments were conducted at the different pH levels (pH 2, 3 and 4). The desired concentrations of Cr were prepared by diluting the standard solution with deionized water. The solution pH was adjusted by adding hydrochloric acid (HCl) or sodium hydroxide (NaCl). The samples were mixed using a rotary shaker at 100 rpm for 24 hr. After the reaction was completed, the liquid phase was separated from the solution using a 0.45 μm PTFE syringe filter (Millipore, USA). The Cr concentration and pH were measured using inductively coupled plasma optical emission spectroscopy (ICP-OES, Optima 2000DV, Perkin-Elmer) and a benchtop pH meter (Orion VERSA 5 star), respectively. Subsequently, the residual PEI-silica nanoparticles, retrieved by rotary evaporation to preserve the adsorbed ions, were analyzed by XPS. In addition, an equilibrium adsorption test of pure nanosilica prior to the synthesis of the nanoparticles using PEI was also conducted to confirm the adsorption capacity of only silica for chromate.

For quantifying the adsorption capacity of the PEI-silica nanoparticles, the equilibrium adsorption of chromium was analyzed by the Langmuir adsorption isotherm model, which is shown in Eq. (1)^53^:

1 $$q=\frac{{q}_{m}{K}_{a}C}{1+{K}_{a}C}$$

where *q*_*m*_ is the maximum mass adsorbed under saturated conditions per unit mass of adsorbent (mg/g). *K*_*a*_ is an empirical constant with units of the inverse of concentration *C* (L/mg).

### Computational Methods

We performed first-principles calculations based on density functional theory using the Vienna Ab-initio Simulation Package (VASP)^58^. The general gradient approximation (GGA) was adapted for exchanging the correlation functional, and the pseudopotential was parameterized under projector augmented wave (PAW) scheme by Perdew-Burke-Emzerhof (PBE)^59–61^. The energy cut-off on a planewave basis was 500 eV, and the force criteria for structure optimization was 0.01 eV/Å. We adopted the DFT-D3 method with Becke-Jonson damping to include van der Waals interactions^62,63^. The lattice was set to 25 × 20 × 15 Å to avoid inter-cell interactions. We analyzed the amount of charges on each atom by using bader charge analysis^64^. All configurations obtained from the theoretical calculations were drawn by using VESTA^65^.

### Data availability

All data generated or analyzed during this study are included in this published article.

## Electronic supplementary material

Supplementary Information
