# Supplementary material for: Chromium removal from aqueous solution by a PEI-silica nanocomposite
Source: Sci Rep. 2018 Jan 23;8:1438. doi: 10.1038/s41598-018-20017-9 (PMC5780388; doi:10.1038/s41598-018-20017-9)
Supplement: Supplementary file 1 — Supplementary Information [file 41598_2018_20017_MOESM1_ESM.pdf]

Supporting Information

Chromium removal from aqueous solution by a PEI-silica nanocomposite

Keunsu Choi[1,†], Soonjae Lee[2,†], Jin Ock Park[3,†], Jeong-Ann Park[4], So-Hye Cho[3,5], Seung Yong Lee[3,5,*], Jun Hee Lee[1,*] and Jae-Woo Choi[4,6,*]

[1] School of Energy and Chemical Engineering, Ulsan National Institute of Science and Technology, Ulsan 44919, Republic of Korea

[2] Department of Earth and Environmental Sciences, Korea University, 145, Anam-ro, Seongbuk-gu, Seoul 02841, Republic of Korea

[3] Materials Architecturing Research Center, Korea Institute of Science and Technology, Hwarang-ro 14-gil 5, Seongbuk-gu, Seoul 02792, Republic of Korea

[4] Center for Water Resource Cycle Research, Korea Institute of Science and Technology, Hwarang-ro 14-gil 5, Seongbuk-gu, Seoul 02792, Republic of Korea

[5] Division of Nano & Information Technology, KIST School, Korea University of Science and Technology, Hwarang-ro 14-gil 5, Seongbuk-gu, Seoul 02792, Republic of Korea

[6] Division of Energy & Environment Technology, KIST School, Korea University of Science and Technology, Hwarang-ro 14-gil 5, Seongbuk-gu, Seoul 02792, Republic of Korea

* corresponding. patra@kist.re.kr (S.-Y. Lee), junhee@unist.ac.kr (J. Lee), plead36@kist.re.kr (J.-W. Choi).

[†] These authors contributed equally to this work

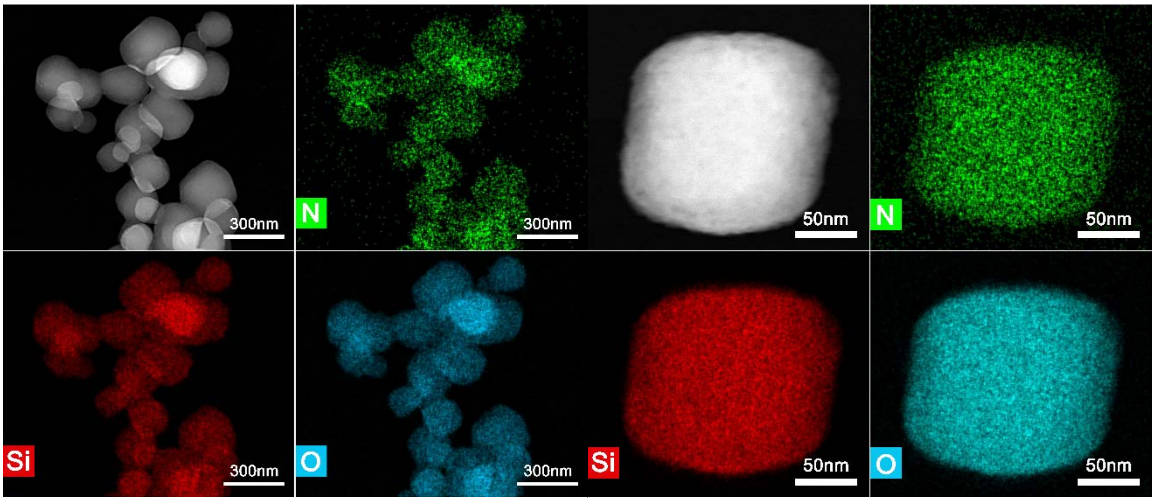

Figure S1. EDS mapping results showing uniformity of N atom distribution.

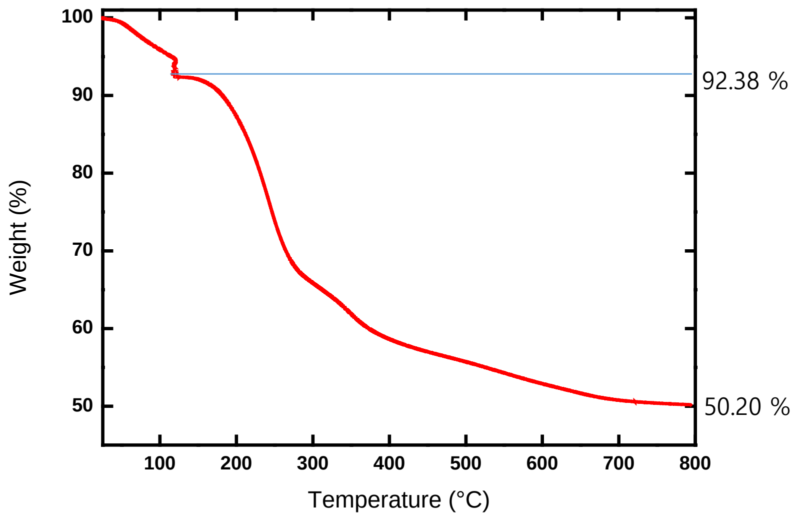

Figure S2. TGA curve in ambient air. Heating rate: 10 $^{o}C\ min^{-1}$. The sample was hold at 120 ^{o}C for 0.5 hr. SDT Q600 (TA instrument) was used.

Exp. S1. Regeneration and reuse of PEI-silica nanoparticle. In order to make the Cr(VI) removal method using PEI-silica nanoparticle more environment friendly and

economical, regeneration and reuse are very important aspect. To validate the reusability of PEI-silica nanoparticle, regeneration experiment was conducted with the PEI-silica nanoparticles used for the Cr(VI) removal by following procedure. The chromium adsorption test was conducted in 50 mL a conical tube containing 0.02 g of PEI-silica nanoparticles by adding 50 mL of chromium solutions with concentrations of 50 mg/L. After reaching equilibrium state, the solution was decanted leaving only the PEI-silica nanoparticles in a conical tube using centrifuge (4000 rpm for 20 min; VS-400, Vision Scientific Co., LTD, Daejeon, Korea). These adsorbents reacted with 5% sulfuric acid solution for 1 h, and then this solution was removed from the conical tube. The regenerated PEI-silica nanoparticles were washed three times with deionized water for using in a re-adsorption test under same conditions. The regeneration tests were repeated five times.

Result of the successive regeneration and reuse of PEI-silica nanoparticles for the Cr removal is shown in Figure S3. The regenerated nanoparticles sustained the Cr removal during the five-successive regeneration and reuse. The removal capacity was maintained for three times of reuse, but it decreased from the fourth reuse. The decrease of the Cr(VI) removal efficiency can be regarded as the influence of the decrease of the reactivity upon reuse and the decrease of the material recovery during regeneration. Due to the nano-sized PEI-silica, it was difficult to recovery the adsorbent completely. The possibility to regenerate the reactive site of PEI-silica nanoparticle was confirmed. The improvement of the adsorbent recovery can be attributed to the increase of the regeneration efficiency by immobilizing PEI at the macromolecular size.

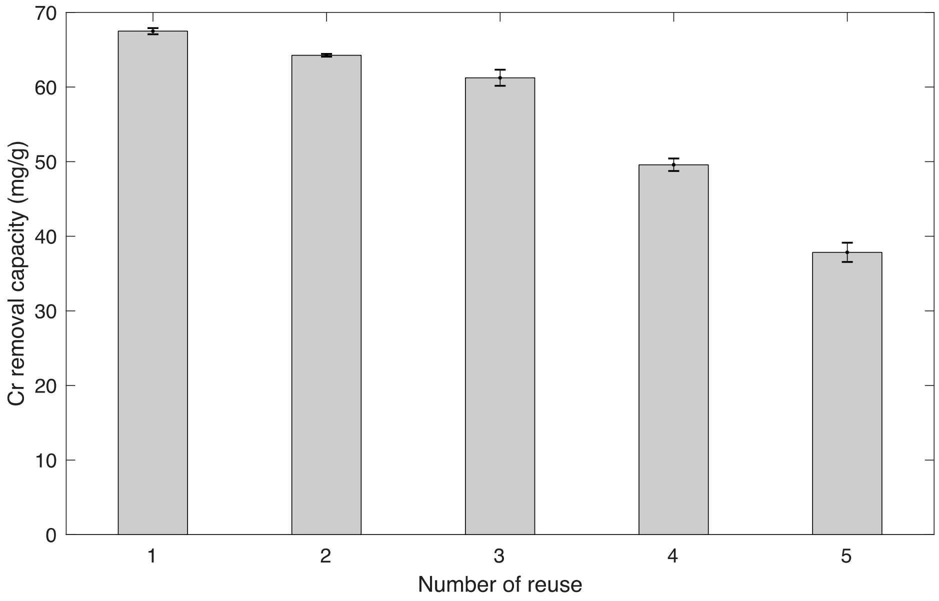

Figure S3. Successive chromate removal using regenerated PEI-silica nanoparticle.
